# A based *Cistanche deserticola* polysaccharide functional-nanoparticle delivery system for effective oral vaccine to facilitate both systemic and mucosal immunity through enhancing oral delivery

**DOI:** 10.1016/j.mtbio.2025.101939

**Published:** 2025-05-31

**Authors:** Jin He, Tianyu Zhu, Lin Yu, Ningning Mao, Xuanqi Lu, Xiaofeng Shi, Xiangwen Deng, Yang Yang, Deyun Wang

**Affiliations:** College of Veterinary Medicine, Nanjing Agricultural University, Nanjing, 210095, PR China

**Keywords:** Dendritic fibrous nano-silica, Oral vaccine, *Cistanche deserticola* polysaccharide, Systemic and mucosal immunity

## Abstract

Oral vaccines received great interest in preventing of global pandemics due to the ability to facilitate both systemic and mucosal immune responses. However, the enzymatic degradation, low permeability of mucus barrier, and lack of effective and safe mucosal adjuvants continue to remain significant challenge for oral vaccine. Herein, we developed a novel delivery system based on nanoparticles functionalized by plant polysaccharides to address the above challenges. Firstly, the *Cistanche deserticola* polysaccharide (CDP) functional-dendritic fibrous nano-silica (DFNS) nanoparticles (CDP-DFNS) were prepared. Encouragingly, the CDP-DFNS with prominent physicochemical and structural characteristics showed high cellular uptake and lead to a high transmembrane transport and intestinal epithelium permeability of antigen. Furthermore, CDP-DFNS significantly induced the antigen internalization and activation of dendritic cells after transport across epithelial cells. In addition, the in vivo experiment results reveled that CDP-DFNS was efficiently facilitated both antigen-specific systemic and mucosal immunity. In conclusion, CDP-DFNS represents a promising candidate for oral vaccine delivery, offering a unique combination of carrier and adjuvant properties. However, further research is needed to evaluate its efficacy in larger animal models and human clinical trials to confirm its translational potential, and fully establish its potential as a next-generation oral vaccine platform.

## Graphical abstract

**Figure:**
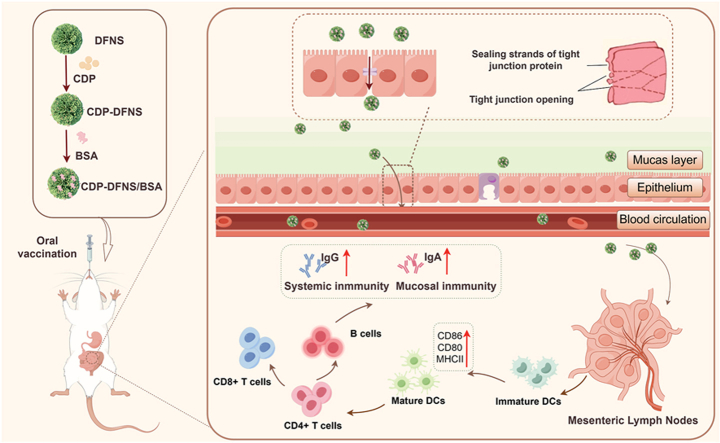

## 1 Introduction

Many microbial pathogens (such as SARS-CoV-2, HIV, and influenza) are entering body via respiratory, gastrointestinal and urinogenital mucosa [1]. It is estimated that 70 % pathogens invade the body through mucosal surfaces due to their large exposed area, thinner and more permeable mucosal membrane barriers compare to skin [2,3]. Thus, mucosal surface as the first-line of defense, an effective strategy which can promote mucosal immune response to protect against pathogens is desirable and attracting increasing attention. When administered directly to mucosal surfaces, mucosal vaccines can induce effective immune responses at the mucosal site and other remote mucosal sites by a common mucosal immune system [4,5]. Oral vaccination is a promising alternative for production of both mucosal antigen specific antibodies (IgA) and systemic antibodies (IgG), with several advantages including easy administration, reduced production costs, and limiting risk of infections [6,7]. However, oral vaccine still remains an onerous challenge limited by the gastrointestinal (GI) tract environment (acidic gastric pH, and proteolytic enzymes) which would lead to its degradation, and biological barriers (mucus layer and tight epithelial cellular junctions) with resulting in a lower uptake of the antigen by the gut and associated lymphoid tissue (GALT) [8,9]. Hence, it is necessary to develop an effective vaccine delivery system that is expected to protected antigen from harsh GI environment, efficiently transport antigen across mucus barrier, activate immune cells, sufficiently induce a strong immune response.

In recent years, various strategies have been developed to solve these aforementioned problems, such as nanotechnologies and new biomaterials [10]. While poor endogenous nature and inefficient endocytosed by the antigen-presenting cells (APCs) still lead to a bottlenecks of oral vaccine development [8]. Mesoporous silica nanoparticles (MSNs) with unique morphological peculiarity and distinct physicochemical features have attracted extensive attentions as oral vaccine carriers. MSNs possessed thermal and chemical stability can protect vaccines from enzymatic degradation in GI [11]. In addition, the unique central-radial pore structures with large pore size and surface area of MSNs provide an efficient degree of antigen, as well as controlling the antigen release [12]. Additionally, MSNs were reported their ability of opening the tight junctions between intestinal epithelial cells and promoting protein absorption [13]. Compared with conventional MSNs, dendritic fibrous nano-silica (DFNS) have the 3D dendritic superstructures with large pore and surface area, and easily functionalized surface, making it has outstanding drug loading ability [14]. Furthermore, DFNS with rough texture similar to virus-like particles can induce effective interaction with cell via mechanical friction, which lead to a higher cellular uptake [15]. Recent studies have shown that DFNS can produce a storehouse to slow and release antigen sustainably, strengthening the combination with antigen presenting cells (APCs) to stimulate a long-term and strong immune responses [16,17]. Admittedly, DFNS with self-adjuvanticity with self-adjuvanticity candidates for enhancing efficacy of oral vaccines.

*Cistanche deserticola* Y. C. Ma (CD), also called as “desert ginseng”, has been used in China for centuries as tonic food and traditional Chinese herbal medicine, firstly recorded in the Shennong's Classic of Materia Medica [18]. Many pharmacological researches have found that CD possessed many pharmacological functions such as anti-tumor, anti-oxidant, anti-fatigue, anti-inflammatory, hepatoprotection, and immunomodulatory [[19], [20], [21], [22]]. *Cistanche deserticola* polysaccharide (CDP), the main bioactive components of CD, have been demonstrated to have various bioactivities including anti-aging, neuroprotective, anti-inflammatory, intestinal flora regulatory functions and immunomodulatory [[23], [24], [25]]. Additionally, recent studies showed that CDP can stimulate dendritic cells (DCs) activation and elicit antigen-specific humoral and cellular responses, even contribute to facilitate cellular immune responses against influenza vaccine in mice [[25], [26], [27]].

**Scheme 1:**
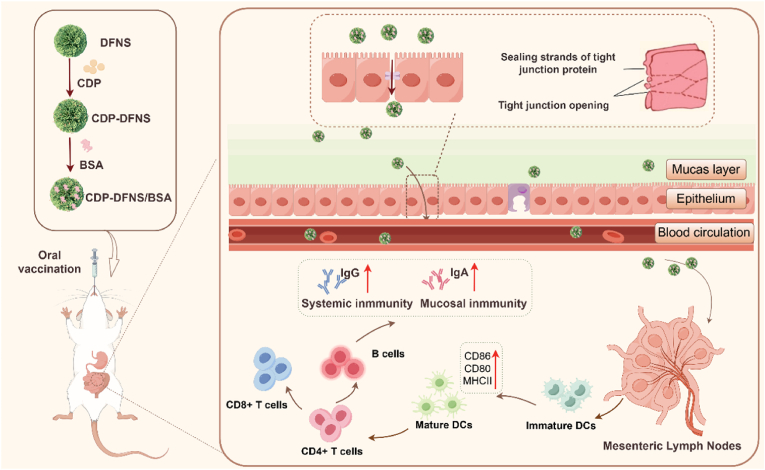
Schematic illustration of the preparation process of CDP-DFNS/BSA, and the induction of both mucosal and systemic immune responses by oral delivery of vaccine.

Our previous study showed that CDP-functional DFNS nanoparticles (CDP-DFNS) represent a novel combination of DFNS with a bioactive polysaccharide, offering enhanced immune activation and mucosal delivery relative to DFNS [28]. In this study, we further demonstrate the feasibility and elucidate the mechanism of CDP-DFNS as an oral vaccine delivery system (Scheme 1). The physicochemical and structural characteristics of CDP-DFNS were systematically characterized, then the cellular uptake, epithelial cell transport, retention and distribution in GI, intestinal villi and mesenteric lymph nodes (MLNs) uptake were investigated. Subsequently, the effect of nanoparticles passing through epithelial cells on the function of mouse bone marrow-derived dendritic cells (BMDCs) was studied subsequently. Then we combined CDP-DFNS and model antigen bovine serum albumin (BSA) to form oral vaccine, and orally immunized mice to investigate the systemic and mucosal immune responses. We anticipate that this study could provide the evidence of CDP-DFNS as a promising candidate of a novel oral vaccine delivery.

## 2 Materials and methods

### 2.1 Materials

The tetraethoxysilane (TEOS), sodium salicylate (Nasal), cetyltrimethylammonium bromide (CTAB), FITC-conjugated BSA, Rhodamine B isothiocyanate (RITC) and cell counting kit-8 (CCK8) were purchased from Solarbio Science & Technology Co., Ltd (Beijing, China). The 1-Ethyl-3-(3-dimethylaminopropyl) carbodiimide (EDC·HOL), N-Hydroxy succinimide (NHS), and 3-aminopropyltriethoxysilane (APTES) were acquired from Shanghai Macklin Biochemical Technology Co., Ltd (Shanghai, China). The Cy5.5 were purchased from Proteintech Group, Inc (Wuhan, China). The anti-CD80-FITC, anti-MHCII-APC, anti-CD11c-PE/CY7, anti-CD86-PE, anti-CD3e-FITC, anti-CD8a-APC, and anti-CD4-PE/CY7 were obtained from Thermo Fisher Scientific (Waltham, Massachusetts, USA). The BSA and HRP goat anti-mouse IgA were obtained from Sigma-Aldrich Co. (MO, USA). The IL-6 and TNF-α ELISA kit were pruchased from Metasciences Biotech Co., Ltd. (Nanjing, China). HRP goat anti-mouse IgG, IgG1, IgG2b, and IgG2a were purchased from ABclonal Biotechnology Co., Ltd. (Wuhan, China). All other reagents were analytically pure.

### 2.2 Cell lines and animals

Caco-2 cells were obtained from Yizefeng Biotechnology Co., Ltd (Shanghai, China), and cultivated in DMEM medium supplemented with 10 % fetal bovine serum and 1 % penicillin and streptomycin. The BMDCs were harvested according the previous reported method, and cultivated in RPMI-1640 medium containing 10 % fetal bovine serum and 1 % penicillin and streptomycin [29].

6-week-old female BALB/c mice were provided by the Comparative Medicine Centre of Yangzhou University. All animal experiments were approved by the Nanjing Agricultural University IACUC (No.2011BAD34B02), and performed in accordance with the guide for the care and use of laboratory animals.

### 2.3 Extraction and purification of CDP and characterization analysis

The method of extraction and purification of CDP was accorded the previous reported method [30]. In brief, CD was purchased from Inner Mongolia Alxa Cistanche Group Co., LTD, sourced from the Alxa region of Inner Mongolia. The raw material was processed using ethanol reflux to remove pigments and fats, followed by enzymatic digestion to eliminate cellulose, starch, and other impurities. Subsequently, crude polysaccharides were extracted through water extraction and alcohol precipitation. The extract was then dialyzed, and the precipitate was collected and purified via gel chromatography to obtain CDP, and detected by the phenol sulfuric acid method. The solubility of CDP was conducted in accordance with the Chinese Pharmacopoeia (2010 Edition). Specifically, 100 mg of the sample was mixed with a certain amount of water. The mixture was vigorously shaken for 30 s every 5 min at room temperature, and the dissolution process was observed over 30 min until the sample was completely dissolved. And the relative molecular mass parameters of CDP were determined by gel chromatography combined with differential angle laser scattering systems [31].

### 2.4 Synthesis and characterization of nanoparticles

DFNS was synthesized using CTAB as template and Nasal as structure-directing agents following our previously reported method [28]. Briefly, 2.85g of CTAB and 0.45g of TEA were dissolved in 200 mL distilled water and hydrolyzed for 3 h under stirring at 80 °C. Then 0.75g of Nasal was put into for reaction another 3 h. Subsequently, 28 mL of TEOS was added to the reaction solution. After 3 h of reaction, the nanoparticle product was collected after centrifugation and wash several times. The DFNS was obtained after removing template by calcining at 600 °C for 6 h.

Next, DFNS were dispersed 50 mg/mL in toluene containing 1.5 mL of APTES, and the mixture was reacted for 4 h. The DFNS-NH_2_ nanoparticles were obtained after centrifugation and wash several times. For preparation of CDP-DFNS, CDP was firstly activated carboxyl by co-reaction with of NHS and EDC·HOL, and subsequently reacted with DFNS-NH_2_ for 12 h. The CDP-DFNS products were centrifuged and washed several times.

For DFNS/BSA and CDP-DFNS/BSA nanoparticles preparation, the 50 mg of DNFS and CDP-DFNS were dispersed in distilled water containing 50 mg of BSA, and stirred 4 h at 4 °C After removing superfluous BSA by centrifugation and wash several times, DFNS/BSA and CDP-DFNS/BSA were collected and stored at 4 °C.

The particle morphology was verified with transmission electron microscope (TEM, HT-7800, Hitachi, Japan) and scanning electron microscope (SEM, QUANTA 250 FEG, ThermorFisher, USA). The Zetasizer Nano-Zeta potentiometer (Nano ZS90, Malvern, UK) was employed to investigate the size, zeta potential and polydispersity index (PDI) of nanoparticles. The XRD patterns of samples at a 2θ angle of 5–45° were conducted with the scanning speed of 4°/min on an X-ray diffractometer (SmartLab, Rigaku, Japan) with a Ni-filtered Cu-Ka radiation. The DSC curves of samples were recorded by a differential scanning calorimetry analyzer (DSC3, Mettler Toledo, Switzerland) from 50 to 350 °C rate of at 10 °C/min. The element composition of nanoparticles was measured and analyzed by field emission transmission electron microscope (2100F, JEOL, Japan).

### 2.5 Preparation of fluorescently labeled nanoparticles

100 mg of RITC was dissolved in 2 mL of anhydrous ethanol, then 100 μL of APTES was added in the above solution and stirred for 24 h away from light to obtain an anhydrous ethanol solution of RITC-APTES. Then 40 mg of DFNS and CDP-DFNS nanoparticles were dispersed in 4 mL of anhydrous ethanol, with addition of 200 μL of RITC-APTES solution for another 24 h reaction. RITC-NPs were obtained after washing several times with anhydrous ethanol until the supernatant clear and drying.

### 2.6 Cytotoxicity assay and cellular uptake

Caco-2 cells were seed in 96-well plates with a density of 1 × 10^5^ cells/mL and co-cultivate with different concentration CDP, DFNS, and CDP-DFNS for 24, 48, and 72 h. The cell viability was detected by using CCK-8 kit. And the cytotoxicity of endocytic inhibitors was detected after 30 min co-cultivation.

For the measurement of cellular uptake *in intro*, Caco-2 cells were plated in 12-well plates (1 × 10^5^ cells/mL) and cultivated for 48 h. The supernatant was discard, then replaced with RITC-NPs (50 μg/mL) for 4 h incubation. Then cells were washed several times with PBS and fixed with formalin, then treated with 50 μL DAPI for 10 min. Finally, the cellular uptake was evaluated by a confocal laser scanning microscopy (CLSM, Nikon A1, Japan). The antigen uptake efficiency was investigated under the same procedure, except that DFNS and CDP-DFNS were loaded with FITC-BSA.

To assess the uptake mechanisms, the cells were pre-treated with different specific endocytic inhibitors including dynasore (25 μg/mL), methyl cyclodextrin (Mβ-CD, 100 μg/mL), cytochalasin D (1 μg/mL), filipin (5 μg/mL), chlorpromazine (CPZ, 10 μg/mL), and chloroquine (30 μg/mL) for 30 min [32]. Then cells were washed and cultivated with RITC-NPs for 4 h, and detected by CLSM. Results were showed as relative cellular uptake compared with that in control group with no inhibitor treatment.

### 2.7 Cellular transmucosal mechanism

To better understand the penetration of NPs in intestinal tract, the polarized Caco-2 monolayers model was established. Caco-2 cells were cultured on the inset chambers of Transwell plates (1 × 10^5^ cells/mL) and incubated for 14 days. The transepithelial electrical resistance (TEER) was detected by a volt-ohmmeter of epithelium (Millicell-ERS, Merck, USA) [33]. Then, cells were cultivated with RITC-NPs for 4 h, and stained with FITC- ZO-1 and DAPI. The distribution of tight junction associated proteins and NPs was detected by CLSM.

### 2.8 Penetration of nanoparticles through intestinal villi

The mice were fasted overnight and then oral administrated with RITC-NPs (40 mg/kg). 4 h after administration, mice were sacrificed, their ileum was collected and prepared for bio-TEM analysis.

### 2.9 Distribution of antigen in GIT and MLNs

To further differentiate the oral absorption of antigen in vivo, the RITC- DFNS and CDP-DFNS loaded with FITC- labeled BSA, and FITC-BSA as control group. 4 h after administration, the ileum and MLNs were isolated, and used for frozen section. The distribution of antigen and NPs in intestine and MLNs was measured by fluorescence microscopy.

In addition, the distribution of antigen in the GIT was investigated by in vivo bioluminescence imaging. The antigen labeled with fluorescence Cy5.5 (Cy5.5-BSA), and then was loaded into DFNS and CDP-DFNS. The mice were treated with different formulations at 20 mg/kg. At 1, 2, 4, 6, 8, 12, and 24 h the intestinal tract, MLNs, and major organs were separated, and then imaged by the IVIS imaging system.

### 2.10 Transport of antigen

Caco-2 cells were cultured on the inset chambers of Transwell plates (1 × 10^5^ cells/mL) and cultivated for 14 days, CDP, DFNS and CDP-DFNS loaded with FITC-BSA were added in the apical compartment. After 6 h cultivation, the medium in the basal compartment was collected, and the fluorescence intensity (FITC, 485/515 nm) was detected using microplate reader of multi-wavelength measurement system (Enspire, PE, USA). The calibration curves which present the amount of antigen transferred across monolayer was established and applied for calculation. The result of antigen transport efficiency was presented as relative transport compared with that in control. And the permeability coefficient (*P*app) was calculated according to the following equations:

$$P\text{app}=dQ\;/\left(dt\times A\times C_{0}\right)$$

where dQ/dt is the transfer volume of antigen per unit time, A is the monolayer area, C_0_ is initial concentration of antigen in the apical compartment.

### 2.11 Cellular uptake and BMDCs activation

To investigate whether NPs which transported across the intestinal epithelial cells activate DCs, the mature BMDCs were seeded in the basolateral compartment (1 × 10^6^/well) and the effect of NPs on DCs function was determined. After 8 h cultivation and balance, CDP, DFNS and CDP-DFNS loaded with FITC-BSA were added in the apical compartment. After 16 h culture, BMDCs were collected, washed with PBS, and stained with anti-CD11c-PE/CY7. The FITC^+^ DCS were detected by flow cytometry (Flow Cytometry Standard, FCS, GCMS-QP20, Beckman, USA). Besides, after 30 h culture, BMDCs were harvested and stained with anti-CD80-FITC, anti-CD11c-PE/CY7, anti-CD86-PE and anti-MHCII-APC. The expression of costimulatory molecules CD80, CD86, and MHCII on DCs was measured by FCS. And the supernatant in the basolateral compartment was collected for ELISA detection of IL-6 and TNF-α.

### 2.12 Immunization study

BALB/c mice (6-week-old) were randomly divided into 5 groups (n = 6): and oral immunized pre mouse on days 1–3, 14 and 21 with following groups: Control (PBS), BSA (free BSA 100 μg), CDP (CDP 100 μg + free BSA 100 μg), DFNS (DFNS/BSA 500 μg), and CDP-DFNS (CDP-DFNS/BSA 500 μg). The immunization schedule and dose were determined based on previous studies [7,34]. On days 28 and 42, blood was collected for hematological analysis and separate serum, and spleen were gathered to evaluation of activation of T cells and lymphocytes proliferation, the intestinal, lung, and genital flushing fluid were obtained by rinsing the duodenum, lung, uterus and vagina with PBS, and heart, liver, lung, spleen, and kidney were collected for histology evaluation. The biochemical indicators in serum and were detect.

On day 21, the spleen was gathered from four mice per group, and then homogenized to obtain lymphocytes. The lymphocytes were plated in 96-well plates (1 × 10^5^/mL), then treated with PHA (5 μg/mL) and LPS (10 μg/mL) for 48 h. The lymphocytes proliferations were evaluated by CCK-8 kits. Other lymphocytes were seeded in 24-well plates (1 × 10^5^/mL) restimulated with antigen BSA (50 g/mL) and subsequently cultivated for 48 h. Lymphocytes were collected after wash with PBS, and stained with anti-CD3e-FITC, anti-CD8a-APC, and anti-CD4-PE/CY7 antibodies. The ratio of CD4 and CD8 of T cells was detected by FCS. In analysis of FCS data, in the untreated cell population (BD in Fig. 5F), live lymphocytes were first gated. Then, CD3^+^ cells were identified from the single-stained control cells treated with CD3 anti-CD3e-FITC antibody. Subsequently, CD3^+^CD4^+^ and CD3^+^CD8^+^ cell populations were gated in the experimental group.

To investigated the immune response in vivo, levels of BSA-specific IgG and its subtypes (IgG1, IgG2a, and IgG2b) in serum and IgA in different flushing fluid were determined by ELISA. And levels of cytokines IL-6 and TNF-α were determined by ELISA kits [28].

### 2.13 Hemolysis assay

The blood samples were collected from untreated mice, and red blood cells (RBCs) were obtained following centrifugation and wash. The RBCs were diluted in 2 % RBCs solution (v/v) with sterile normal saline. Then 2 mL RBCs solution was mixed with 2 mL sterile normal saline (negative control group), deionized water (positive control group) and different concentrations of DFNS and CDP-DFNS for 4 h incubation. Then cells were centrifuged, photographed, and the absorbance of the supernatant at 541 nm wavelength was measured. The hemolysis rate was evaluated by following equation:

$$\text{hemolysis}\;\text{ratio}\;\left(\%\right)=\left(A_{\text{test}}-A_{\text{negative}}\right)/\left(A_{\text{positive}}-A_{\text{negative}}\right)\times 100\%$$

where A_test_, A_negative_, and A_positive_ is the absorbance of test groups, negative control group, and positive control group, respectively.

### 2.14 Statistical analysis

Data were presented as mean ± the standard deviation (SD). All statistical tests of data were executed by SPSS software package (version 21, IBM). Significance of differences in groups were analyzed by two-way ANOVA with Duncan's multiple range test. Statistical significance is presented as ∗*p* < 0.05, ∗∗*p* < 0.01, ∗∗∗*p* < 0.001.

## 3 Results and discussion

### 3.1 Characteristics of nanoparticles

**Scheme 2:**
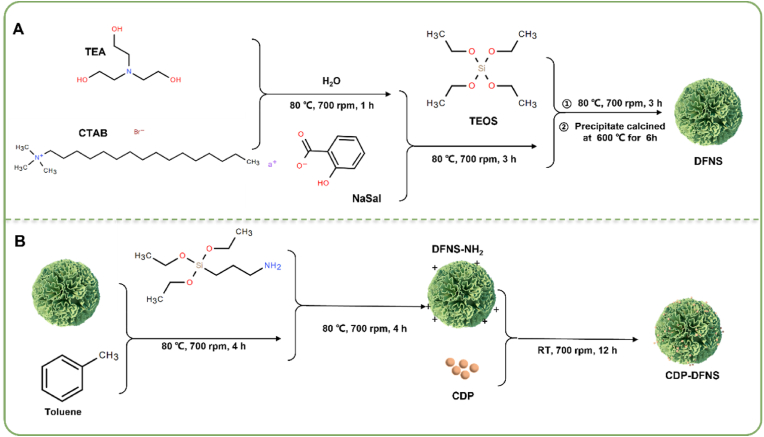
Schematic illustration of the preparation process of DFNS (A) and CDP-DFNS (B).

In this study, DFNS nanoparticles were prepared through a facile and economical silica constructing program by a one-pot method, utilizing TEOS as the main silica source, NaSal and CTAB as constructure-directing agents, and TEA as a catalyst. In this method, CTAB associated to form micelles in aqueous solution of TEA, following the addition of auxiliary template NaSal to assembled with CTAB to form a silica-based nanoparticles with a dendritic pore network (Scheme 2). Then TEOS was added and sedimented to form a silicon skeleton [35,36]. Finally, the DFNS with central radial pore structures and tunable particle size were obtained after removing template trough high-temperature calcination.

The molecular weight parameters of CDP are detailed in Table S2, where Mw and Mw/Mn are approximately 132.124 kDa and 2.625, respectively. And the solubility of CDP was 152 mg/mL. Combined with FITR results (Fig. S1), CDP is a novel homogeneous acidic polysaccharide from CD. After amino modification, CDP-DFNS were successfully prepared through chemical bond reaction within the amidogen in DFNS and carboxyl group in CDP.

**Fig. 1:**
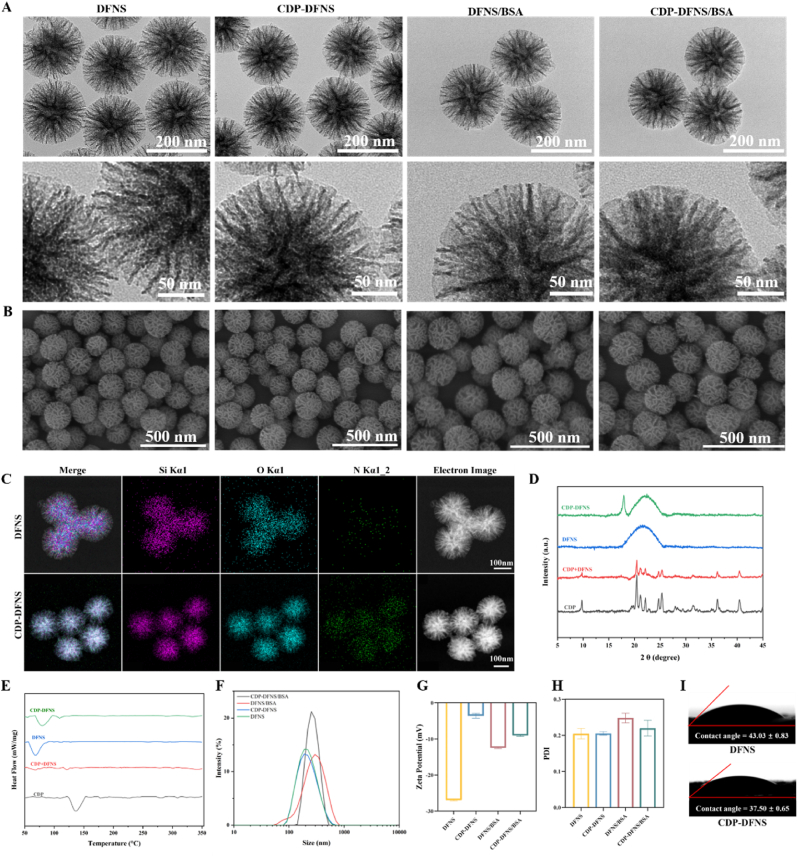
**The characterizations of nanoparticles.** The TEM (A) and SEM (B) images of DFNS, CDP-DFNS, DFNS/BSA, and CDP-DFNS/BSA. (C) EDS mapping of DFNS and CDP-DFNS, indicating the distribution of Si, N, and O. The XRD patterns (D) and DSC curves (E) of CDP, DFNS, and CDP-DFNS. The size (F), zeta potential (G), and PDI (H) of DFNS, CDP-DFNS, DFNS/BSA, and CDP-DFNS/BSA. (I) Initial contact angles of DFNS and CDP-DFNS. Results were presented as means ± SD (n = 3).

As shown in TEM images (Fig. 1A), DFNS and CDP-DFNS both were spherical monodispersed uniform nanoparticles with a distinguishable mesoporous structure, consistent with our previous nitrogen adsorption–desorption test [28]. The SEM observation (Fig. 1B) showed that DFNS and CDP-DFNS had a rough surface with plenty of slender curved mesochannels homogeneous. The hydrodynamic diameters of DFNS and CDP-DFNS were 203.3 ± 1.87 and 218.4 ± 5.07 nm, with the PDI of 0.204 ± 0.025 and 0.205 ± 0.009, respectively (Fig. 1F–H). And zeta potential analysis showed that DFNS was −26.96 ± 0.30 mV, whereas CDP-DFNS was −3.57 ± 1.14 mV on account of the amino-modification before grafting CDP (Table S1). In the gastrointestinal tract, mucus is considered a major barrier to the penetration of NPs, hindering both positively and negatively charged NPs. However, when NPs possess a net neutral surface charge, they can overcome this barrier, traverse the mucus layer, and directly interact with intestinal epithelial cells [37]. The morphology and structure of nanoparticles was no obvious change after BSA loading (Fig. 1A and B). The average size of DFNS/BSA and CDP-DFNS/BSA were 246.0 ± 4.55 and 261.5 ± 3.01 nm, were slight larger than pure nanoparticles. And DFNS/BSA and CDP-DFNS/BSA had a zeta potential of −12.43 ± 0.35 and −9.01 ± 0.43 mV, with PDI of 0.248 ± 0.023 and 0.220 ± 0.037 (Fig. 1F–H).

The energy-dispersive X-ray spectroscopy (EDS) elemental mapping results indicated the homogeneous distribution of silicon and oxygen in DFNS, while silicon, oxygen, and nitrogen in CDP-DFNS (Fig. 1C). The presence of N elements exhibited the successful preparation of CDP-DFNS. Fig. 1D revealed XRD patterns of DFNS, CDP-DFNS and CDP, the sharp peaks between 5° and 45° corresponds to crystalline state for CDP. While DFNS present an amorphous silica indicated by the broad peak between 15° and 30° [[38], [39], [40]]. In addition, in XRD spectra of CDP-DFNS, sharp diffraction peak of CDP transformed into broad peak similar to DFNS, suggesting that CDP was confined in an amorphous form in the mesopore in the mesopores. The DSC analysis (Fig. 1E) was consistent with the XRD results, the DSC curves of CDP had an intense and characteristic endothermic peak (137.12 °C), while that of CDP-DFNS were almost amorphous broad peaks with no distinct exothermic peaks. The XRD and DSC results demonstrated that CDP was efficiently loaded into mesoporous of DFNS in amorphous state. Because DFNS possessed the limited nano-scale pore size which led to the transform of CDP crystals to amorphous states [41,42]. To understand the affinity between nanocarriers and the liquid bio-interface, the wettability of DFNS and CDP-DFNS was evaluated using the static drop technique. As descripted in Fig. 1H, the initial contact angles of DFNS and CDP-DFNS were 43.03 ± 0.83 and 37.50 ± 0.65, respectively, suggesting the hydrophilic nature. TEM images in Fig. S2 showed that CDP-DFNS degraded over time and minimal degradation was observed only after 7 days, confirming its protective effect on antigen stability in harsh gastric conditions. Progressive particle aggregation and increased degradation occurred by day 14, effectively mitigating potential safety concerns associated with long-term nanoparticle retention in vivo.

### 3.2 Cellular uptake of nanoparticles

**Fig. 2:**
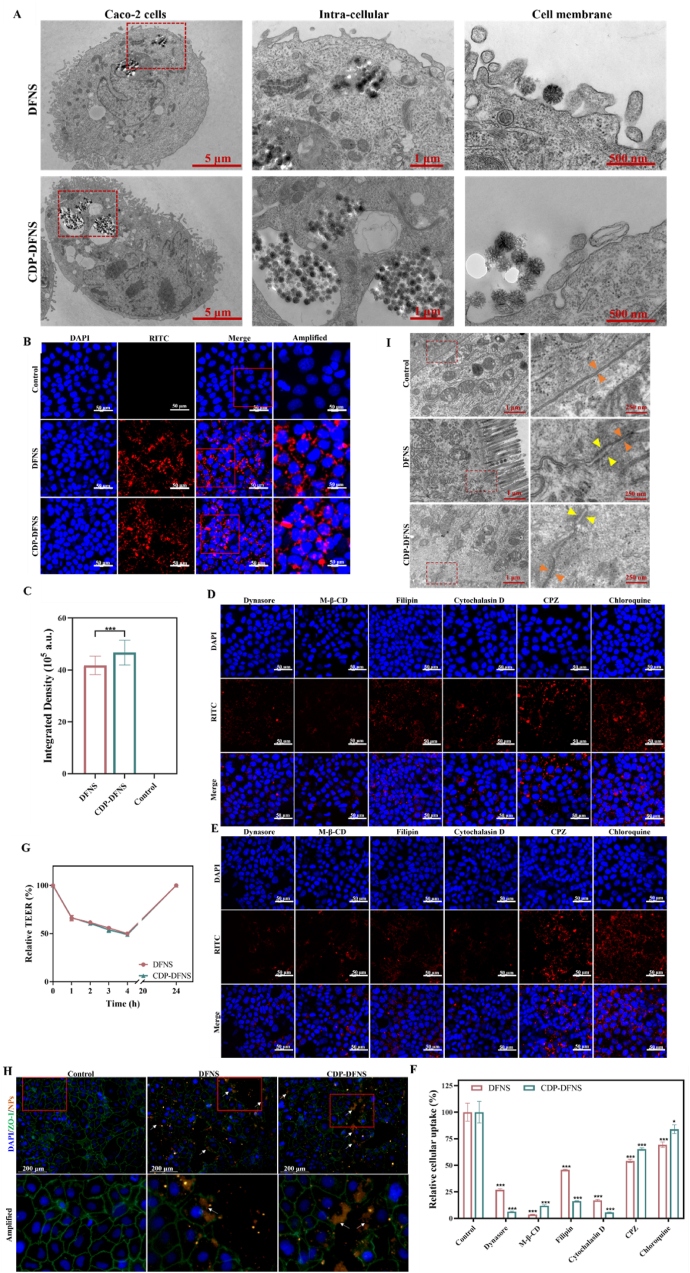
**Cellular uptake and transepithelial delivery of nanoparticles.** (A) Bio-TEM images of the cellular uptake of NPs on Caco-2 cells, scale bar 5 μm,1 μm and 500 nm. (B) CLSM images of the cellular uptake of DFNS and CDP-DFNS, red: RITC-NPs, blue: nuclei of the cells, scale bar 50 μm. (C) The integrated intensity of RITC in CLSM images. (D) The relative cellular uptake of DFNS and CDP-DFNS after incubation with specific inhibitors. CLSM images of the cellular uptake of DFNS (E)and CDP-DFNS (F) after incubation with specific inhibitors, scale bar 50 μm. (G) The TEER value before and after the addition of DFNS and CDP-DFNS. (H) The distribution of ZO-1 between adjacent cells after treating with DFNS and CDP-DFNS, red: RITC-NPs, green: ZO-1, blue: nuclei of the cells, scale bar 200 μm. (I) Bio-TEM images of tight junctions between intestinal epithelial cells, scale bar 1 μm and 250 nm. All results were presented as means ± SD (n = 4). ∗p < 0.05, ∗∗p < 0.01, ∗∗∗p < 0.001. (For interpretation of the references to colour in this figure legend, the reader is referred to the Web version of this article.)

The Caco-2 cells has been widely used to perform experiments that simulate intestinal transport. Thus, RITC labeled DFNS and CDP-DFNS were co-incubated with Caco-2 cells and the epithelial uptake and internalization of NPs were evaluated by bio-TEM and CLSM. Firstly, the cell viability of all formulations was not significantly reduced compare with that of control group with 4 h, 24 h, and 48 h co-incubation, which showed no cytotoxicity and favorable cytocompatibility (Fig. S3). Bio-TEM analysis (Fig. 2A) revealed that both DFNS and CDP-DFNS nanoparticles exhibited extensive membrane adhesion, with multiple contact points established between the rough nanoparticle surfaces and cell membranes. This direct membrane anchoring appears to facilitate subsequent cellular internalization processes. Encouragingly, numerous NPs appeared in the Caco-2 cells. As shown in Fig. 2B and C, DFNS and CDP-DFNS both exhibited strong red fluorescence signals, demonstrating the high efficiency of cell entry. Moreover, CDP-DFNS showed cellular uptake efficiency evidenced by the stronger fluorescence intensity. This phenomenon could be attributed to the strong contact with lipid membrane layers and activation of epitopes on the cells by silica nanoparticles, which is consistent with previous studies [43,44]. Compared with DFNS, the higher uptake of CDP-DFNS may be related to its enhanced hydrophilicity (Fig. S4).

Thereafter, to understand the cellular uptake pathways of nanoparticles, cells were pretreated variety of specific inhibitors, including dynasore, Mβ-CD, filipin, cytochalasin D, CPZ, and chloroquine before co-incubating with DFNS and CDP-DFNS (Fig. 2D–F). Compared with control group, all inhibitors significantly reduced the uptake of DFNS and DFNS to a varying extent. For DFNS, the cellular uptake was significantly reduced about 73.1 %, 45.8 %, 30.6 % after pre-treated with dynasore, CPZ, and chloroquine, suggesting that its endocytosis was associate with the clathrin-mediated endocytosis. Moreover, the decrease of Mβ-CD and filipin on cellular uptake of DFNS was approximately 96.3 % and 55.8 % which was related to caveolae mediated endocytosis. In the presence of cytochalasin D, the cellular uptake of DFNS was reduced to 83 %, implying the micropinocytosis. Similarly, the internalization of CDP-DFNS was sharply decreased in the presence of dynasore, Mβ-CD, filipin, and cytochalasin D of 92.1 %, 99.5 %, 99.0 %, and 98.5 %, respectively, indicating that micropinocytosis, clathrin and caveolae-mediated endocytosis all play critical roles in the cellular uptake process. In conclusion, CDP-DFNS showed more effectively cellular uptake in Caco-2 cells and was complicated referring to multiple pathways.

### 3.3 Paracellular transport through opening tight junction

Tight junctions, which distribute whin adjacent epithelial cells, play important parts in producing adhesion barrier between cells to maintain the integrity of the epithelium [45]. Thus, we established Caco-2 monolayers as the model of the intestinal epithelium and then measured the effect of DFNS and CDP-DFNS on the integrity of the epithelium. The TEER values of monolayers were increased gradually over time and reached in 300 Ω/cm^2^ on day 5, indicating the well integrity of the cell monolayers at this time (Fig. S6) [46,47]. To determine whether nanoparticle transport across cell monolayer through open tight junction, DFNS and DFNS were applied to the apical compartment. The CLSM results reveal that tight junction proteins ZO-1 both in DFNS and CDP-DFNS group was interrupted, discontinuous and obscure (as shown by white arrows), which extremely differ from the continuous ring feature in control group (Fig. 2H). In addition, after treated with DFNS and CDP-DFNS for 4 h, TEER values were significantly decreased to 50–60 % of the initial value (Fig. 2G), which is consistent with the CLSM results. And TEER was recovered again after removing nanoparticles, suggesting that DFNS and CDP-DFNS open tight junctions temporarily and don't damage the structure of the epithelial monolayers.

Subsequently, bio-TEM analysis was showed in Fig. 2I, an opened and diffuse band was observed in intercytoplasmic margins, the gap between the yellow arrows (associated with opened tight junctions) was distinctly wider than that in the orange arrows (associated with closed tight junctions), further demonstrating that DFNS and CDP-DFNS can transport across enterocytes through open tight junctions.

### 3.4 Transmembrane transport of antigen

**Fig. 3:**
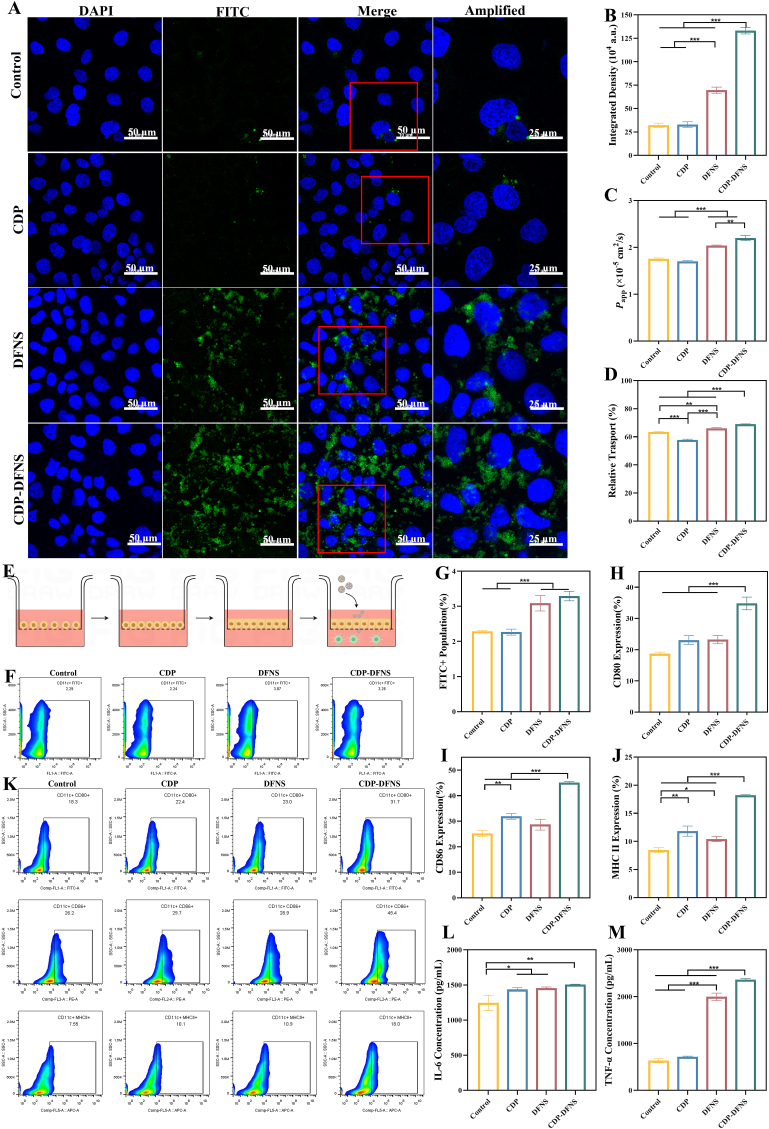
**Transmembrane transport and cellular uptake by BMDCs.** (A) CLSM images of the cellular uptake of antigen on Caco-2 cells, green: FITC-BSA, blue: nuclei of the cells, scale bar 100 and 50 μm. (B) The integrated intensity of FITC in CLSM images. The *P*app values (C) and relative transport for the initial concentrations (D) of antigen in Caco-2 monolayers model. (F–G) Cellular uptake of FITC-BSA-loaded nanoparticles in BMDCs by flow cytometry. Expression of CD80 (H), CD86 (I), and MHCII (J) in BMDCs, and the flow cytometry plots (K). The concentration of IL-6 (L) and TNF-α (M) in BMDCs. All results were presented as means ± SD (n = 4). ∗p < 0.05, ∗∗p < 0.01, ∗∗∗p < 0.001. (For interpretation of the references to colour in this figure legend, the reader is referred to the Web version of this article.)

After oral vaccination, the antigens pass through the intestinal epithelium and are delivered to APCs that located in the lamina propria, which is the first step in initiating the body's immune response [48]. After loading with BSA, DFNS and CDP-DFNS were co-cultured with Caco-2 cells and the antigen cellular uptake and transport were determined. According to Fig. 3A and B, the strongest green fluorescent signal was observed in cell after treatment with CDP-DFNS/BSA, suggesting the highest cellular uptake efficiency of antigen. However, the fluorescence intensity originating from control and CDP group reveled a relatively weak fluorescence intensity. DFNS also showed its excellent promotion of antigen uptake efficiency compare to the non-nanoparticle treated group. The specific interactions were appeared between CDP-DFNS and the receptors and membrane proteins on the cell surface, which facilitate the uptake of nanoparticles and antigens [49].

The transmembrane transport of antigen was also evaluated in the Caco-2 monolayer model. After 6 h co-incubation, DFNS and CDP-DFNS both allowed more antigen transport across the intestinal barrier, showed higher antigen permeability (Fig. 3C) relative transport of the initial concentrations (Fig. 3D), especially CDP-DFNS showed the highest *P*app value. This result was attributed to the ability of nanoparticles to open tight junctions between intestinal epithelial cells.

### 3.5 Cellular uptake and activation of BMDCs

According to Fig. 3E, the mature BMDCs were seeded in the in the basal compartment, and the effect of nanoparticles passing through epithelial cells on the function of BMDCs was studied subsequently. The FCS analysis was showed that DFNS and CDP-DFNS had a higher population of FITC + DCs compared with that of CDP and BSA alone, which is consistent with the result of acting with single BMDCs (Fig. 3F and G) [28]. After internalization, facilitating DCs translate to active state is important for subsequent antigen-specific immune response [50]. As previous study reported that adjuvants can enhance the immune effect by effectively accelerating DCs activation [51]. Therefore, to subsequently understanding whether CDP-DFNS can promote DCs activation, the co-expression of costimulatory molecules CD80, CD86 and MHCII were detected by FCS. As showed in Fig. 3H–K, CDP-DFNS obviously increased the expression of CD80, CD86 and MHCII on BMDCs compare with control group, revealing its potential adjuvanticity, which is consistent with previous study [52,53]. In general, the activated DCs produce and secrete some cytokines such as IL-6 and TNF-α [54,55]. The levels of secretion levels of IL-6 and TNF-α in co-culture supernatant were determined by ELISA. According to Fig. 3L and M, CDP, DFNS, and CDP-DFNS induced a higher secretion of IL-6 compared with that in control group. And it is inspired that DFNS, and CDP-DFNS both improved TNF-α production, whereas that in CDP-DFNS was remarkably higher than that treated with DFNS. In summary, these above results together demonstrated that CDP-DFNS after effectively transport across epithelial cells, can subsequently improve cellular uptake and stimulate DCs activation, potentially increase their immunity-enhancing activity.

### 3.6 Transepithelial permeability and distribution in GI tract of antigens

**Fig. 4:**
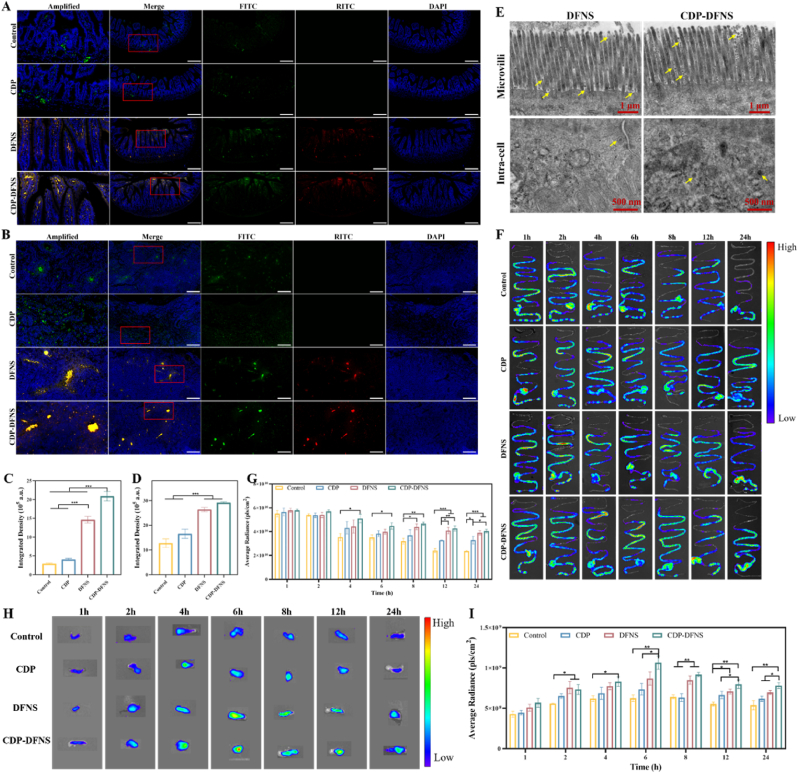
**The transepithelial permeability and biodistribution of nanoparticles.** Fluorescent microscopy images of intestinal villi (A) and MLNs (B) after oral administration for 4 h, scale bar 200 μm. Quantitative analysis of FITC-labeled antigen in intestinal villi (C) and MLNs (D). Bio-TEM images of intestinal epithelial cells, scale bar 1 μm and 500 nm. Fluorescence images of Cy5.5-labeled antigen distribution in GI (F)tract and MLNs (H). Quantitative analysis of Cy5.5-labeled antigen in GI (G)tract and MLNs (I). All results were presented as means ± SD (n = 3). ∗p < 0.05, ∗∗p < 0.01, ∗∗∗p < 0.001.

Given the promising results of CDP-DFNS in cellular uptake and transmembrane transport *in vitro*, the delivery process via intestinal villi and MLNs transport were investigated in vivo. Mice were orally administered with RITC labeled nanoparticles loading with FITC-BSA, and their small intestine and MLNs were collected, sliced and observed using CLMS. In accordance with Fig. 4 A and C, in sharp contrast to control and CDP which showed low FITC fluorescence intensity that distributed in interior of the villus, CDP-DFNS and DFNS were distributed throughout the villus and almost penetrated into the lamina propria with stronger fluorescence signal. Additionally, CDP-DFNS and DFNS were mainly distributed at the lacteal which located in the center of intestinal villi, indicating that the lymphatic transport of nanoparticles and antigen via the chylomicron pathway [56]. In the process of transmucosal transport, the FITC and RITC fluorescence signal were all along overlapped, suggesting that CDP-DFNS possessed high efficiency of intestinal absorption by themselves and subsequently improve transport capacity of the loaded antigens. Furthermore, the bio-TEM study also was performed to investigate the cellular uptake nanoparticles. A large number of nanoparticles aggregating around the microvilli was both observed in DFNS and CDP-DFNS, and some were entered into the intra-cell as the yellow arrowed showed (Fig. 4 E). In contrast, the internalization of DFNS was less than that of CDP-DFNS, which revealed that CDP-DFNS possesses satisfactory intestinal transport characteristic, presenting a promising lymphatic targeting capacity.

**Fig. 5:**
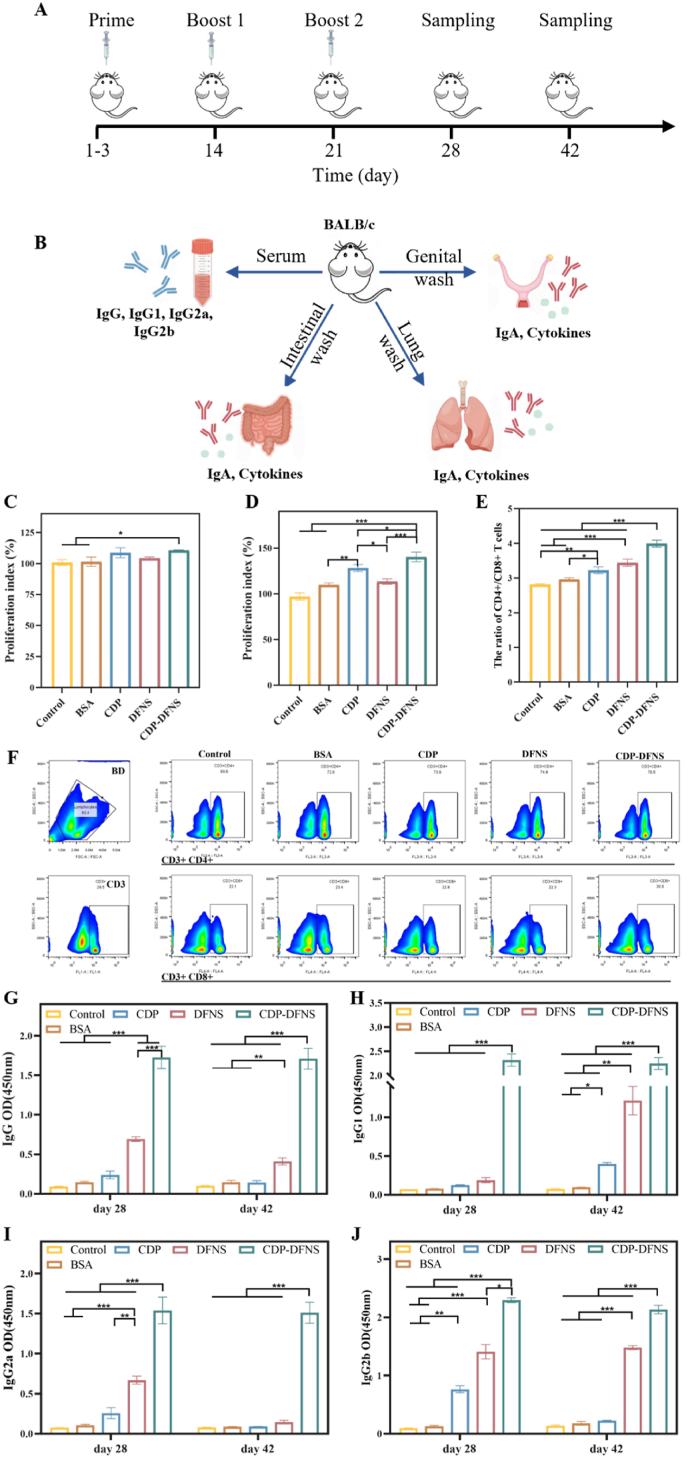
**Lymphocyte differentiation in spleen and antibodies responses in serum.** A schematic of the vaccination procedure (A), and sampling (B). (C) B lymphocyte proliferation and (D) T lymphocyte proliferation induced by LPS and PHA. (E) The ratio of CD4+/CD8+ T cells in spleen on day 28. (F) The representative flow cytometry plots of gating strategy and ratio of CD4^+^ and CD8^+^ T cells. The levels of IgG, IgG1, IgG2a, and IgG2b in serum on days 28 and 42. All results were presented as means ± SD (n = 4). ∗p < 0.05, ∗∗p < 0.01, ∗∗∗p < 0.001.

With the aim of investigating the absorption performances and bio-retention of DFNS and DFNS in intestinal tract, the biodistribution of antigen were evaluated using in vivo imaging system. Mice were orally administered with Cy5.5-BSA, mixture with CDP and Cy5.5-BSA, DFNS and CDP-DFNS loading with Cy5.5-BSA, and then were euthanized at different times to harvest intestinal tract for evaluating the fluorescence intensity. As displayed in Fig. 4F and G, the fluorescence intensity from control and CDP begun to decreased rapidly at 2 h after administration, and almost fluorescence signals then transferred to large intestine at 8 h. Instead of being quickly excreted, for CDP-DFNS, the fluorescence intensity decreased slowly, and were significantly stronger than that of other groups. Meanwhile, DFNS displayed a good bio-retention compare with control group, but the fluorescence signals intestinal tract was lower than CDP-DFNS after 24 h post administration, reflecting that CDP-DFNS can prolong the antigen retention and further provide a sufficient antigen exposure [57]. After 24 h, very faint fluorescence signals were observed in the major organs of control and group, and was little distributed in the liver and kidney of DFNS and CDP-DFNS group (Figure S7). Thus, nanoparticles can influence the antigen distribution and retention.

### 3.7 MLNs targeted delivery of nanoparticles

We then investigated the ability of CDP-DFNS to deliver antigen to MLNs, where adaptive immune responses initiated during oral vaccination [9,58]. The result of immunofluorescence assay showed that the FITC fluorescence intensity of CDP-DFNS in MLNs was obviously higher tang that of control and CDP group (Fig. 4B and D). The similar tendency has been observed in Fig. 4H, the fluorescence signal of CDP-DFNS in MLNs was significantly higher compared with that of other groups. The fluorescence signal of control and CDP group were declined with time after administration for 4 h, while that of CDP-DFNS were peaked at 6 h after administration, and maintained a higher level for a long time, indicating the excellent lymphatic targeting ability, which was beneficial from the efficient absorption by intestine (Fig. 4 I). The extended dwell time of antigen in lymph nodes is beneficial to playing the role of CDP-DFNS as an antigen depot, which can continuously release antigens and provide a longer interaction time between APCs and antigens [59].

### 3.8 Systemic immune responses

In consideration of the outstanding physicochemical and intestinal transport specialty of CDP-DFNS, whether the nanoparticles as oral vaccine delivery system can effectively induce the systemic immunity and mucosal need to be detected. Mice were orally administrated with different formulations at priming three days, and proceed a boost vaccination on day 14 and 21 (Fig. 5A). Following oral vaccination, antigen-presenting DCs in gut mucosa associated lymphoid tissue migrate to migrate to MLNs or the systemic circulation, and interact with T cells in the spleen for further activation of T cells [9]. Firstly, the proliferation of B and T cell in spleen stimulate by LPS and PHA was evaluated by CCK-8 method as previous study described [60]. As exhibited in Fig. 5C and D, after 48 h incubation, CDP-DFNS led to the increased proliferation index of both B and T cell compared to BSA, while CDP also possessed an elevation of in T cells proliferation, but fail to allow a higher B cells proliferation. The ratio of the CD4+/CD8+ T cells is currently identified as the most important indicator for the evaluation of intrinsic immune response, because high ratio of CD4/CD8 T cells are commonly observed in individuals with increased immune capacity ability [61,62]. As shown by the flow cytometry results, CDP, DFNS, and CDP-DFNS all up-regulated the CD4+/CD8+ T cells ratio compared to BSA group (Fig. 5 E), with CDP-DFNS showing the highest ratio among other groups. The DFNS with high loading efficiency of antigen are more easily absorbed by APCs, and increase the antigen cross-presentation to enhance the CD4^+^ T cells activation (Fig. S8A) [53].

To further investigate the humoral immune responses of CDP-DFNS in mice, the levels of systemic BSA-specific antibody in serum on days 28 and 42 were detected by ELISA. More strikingly, the levels of BSA-specific IgG, IgG1, IgG2a, and IgG2b in CDP-DFNS group were increased sharply compared with those in other groups on days 28 and 42 (Fig. 5G–J). However, DFNS also showed higher IgG, IgG1, and IgG2b levels compared to BSA groups on day 42, while higher IgG2a levels on day 28. Taken together, these results demonstrated that CDP-DFNS as oral vaccination delivery system effectively induced strong systemic antibodies immune responses.

### 3.9 Mucosal immune responses

**Fig. 6:**
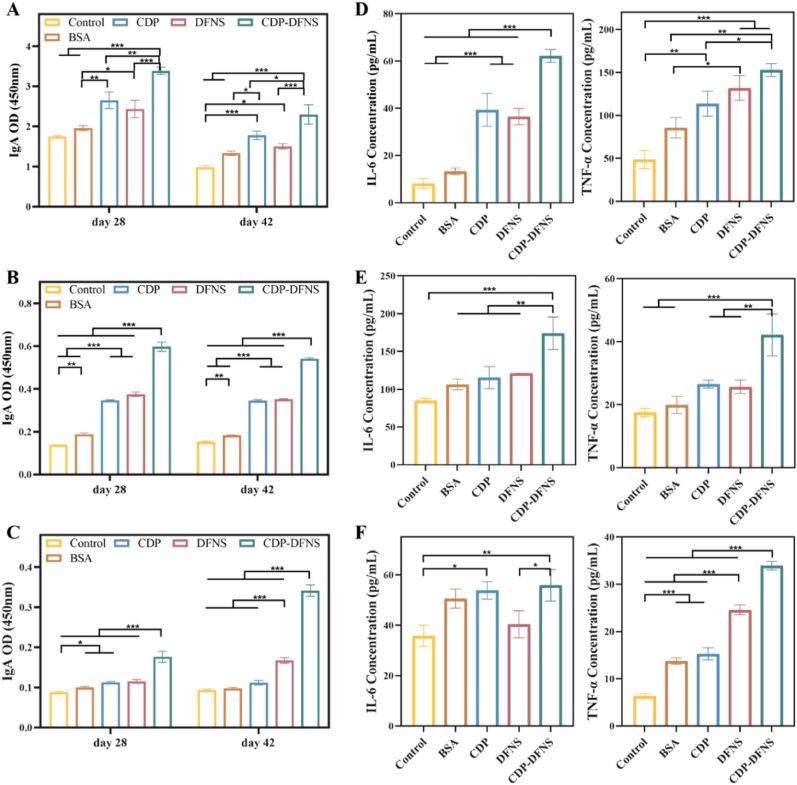
**Mucosal immune responses of CDP-DFNS.** The levels of BSA-specific IgA in intestinal (A), lung (B) and genital (C) flushing fluids on days 28 and 42. The levels of IL-6 and TNF-α in intestinal (A), lung (B) and genital (C) flushing fluids on day 28. All results were presented as means ± SD (n = 4). ∗p < 0.05, ∗∗p < 0.01, ∗∗∗p < 0.001.

The sites of the mucosal system are anatomically separate and independent, but functionally they are part of a “common mucosal system” [9]. This means that antigen presentation and B cell activation at one mucosal site can lead to the secretion of IgA at other distant mucosal sites, collectively defending against pathogen invasion [63]. Following mucosal vaccination, the strongest immune response occurs at the mucosal site directly exposed to the antigen, followed by the surrounding tissues exposed to the antigen [64]. Oral vaccination with appropriate delivery system can induce efficient mucosal immunity in different mucosal tissue due to the “common mucosal immune system” theory [65]. Antigen-specific IgA located on the mucosal surfaces is critical for protecting mucosa from virus and preventing opportunistic infections [66]. To verify whether CDP-DFNS could trigger the mucosal immunity in vivo, the BSA-specific IgA levels in intestinal, lung and genital flushing fluids were examined by ELISA (Fig. 5 B). As shown in Fig. 6 A, in intestinal lavage fluids, significant higher IgA levels on day 28 were observed in CDP, DFNS, and CDP-DFNS compared to BSA group. Notably, CDP-DFNS induced higher IgA levels compared to CDP and DFNS groups on day 28 and 42, on account of the better transmembrane transport and lymphatic targeting characteristics. In addition, CDP, DFNS, and CDP-DFNS facilitated the higher BSA-special IgA levels in lung lavage fluids than BSA on days 28 and 42, while CDP-DFNS led to the highest up-regulated IgA levels among them (Fig. 6 B). The similar tendency of IgA levels was found in genital flushing fluids (Fig. 6C), indicating the feasibility of CDP-DFNS as oral vaccine delivery system to produce effective and long-lasting mucosal immunity. Meanwhile, the cytokines IL-6 and TNF-α in intestinal, lung and genital flushing fluids were also evaluated on day 28. As shown in Fig. 6D–F, both of IL-6 and TNF-α in intestine and lung were enhanced obviously in CDP-DFNS group than that in BSA group. The production of pro-inflammatory cytokine IL-6, which contributes to the recruitment of immune cells, indicating that CDP-DFNS induce immune response through a potentially inflammatory way [67]. Altogether, orally immunized with CDP-DFNS facilitate T and B cells migrate to systemic secondary lymphoid tissues and subsequently to different mucosal tissues, and then accelerated strong both systemic and mucosal immune responses [4].

### 3.10 Safety analysis

**Fig. 7:**
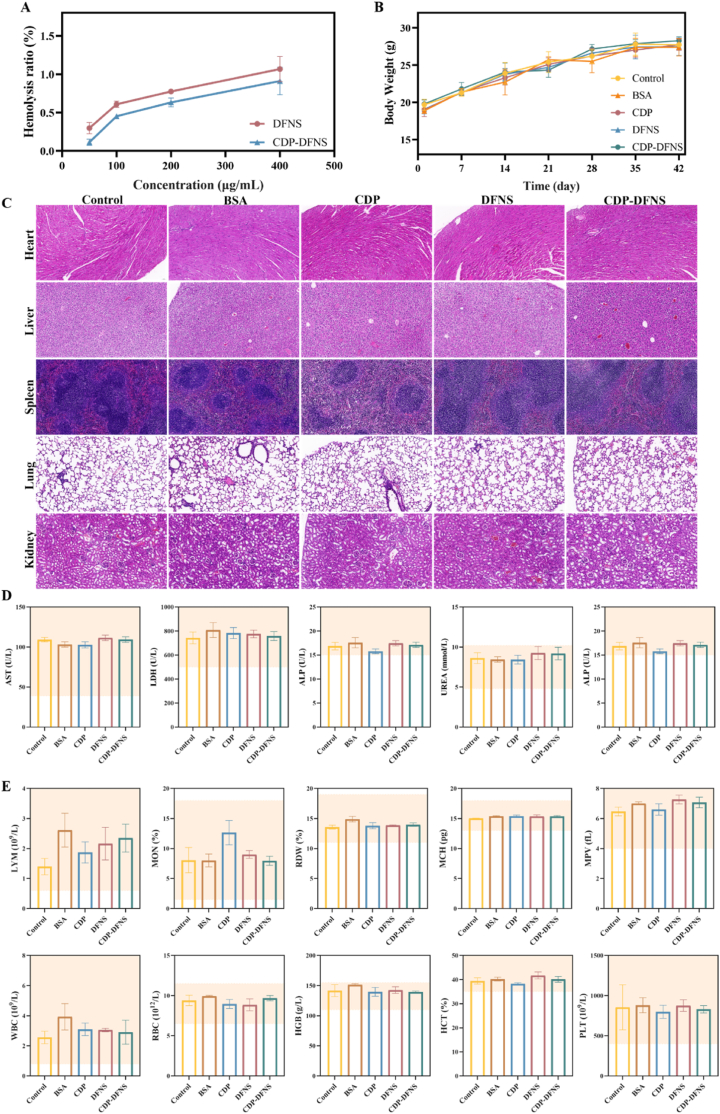
**Safety assay of nanoparticles.** (A)The hemolysis ratio of DFNS and CDP-DFNS in concentration of 50–400 μg/mL. The body weights (B), H&E staining images of the major organs (C), and levels of biochemical (D) and hematology indicators (E) of mice at day 42. All results were presented as means ± SD (n = 4). ∗p < 0.05, ∗∗p < 0.01, ∗∗∗p < 0.001.

Taking into account the potential toxicity of nanoparticles, the biocompatibility of DFNS and CDP-DFNS was explored *in vitro* and in vivo. Hemolysis assay were performed to investigated the hemocompatibility of nanoparticles at the concentration of 50–400 μg/mL. As shown in Fig. 7 A and Fig. S9, the hemolysis ratio of DFNS at the final concentration was 1.06 %, and that of CDP-DFNS even less than 1 %, indicating the compatibility of CDP-DFNS with red blood cells and the safety in blood circulation. Then the toxicity of nanoparticles in vivo was conducted to examine the body weights of animals, histopathological, biochemical and hematological detection. During the experiment period, the body wights of mice in each group showed the same growth trend with control group, and no obvious weight loss and abnormal behaviors was observed. At the last day of experiment, mayor organs (heart, liver, spleen, lung, and kidney) were harvest and conducted for H&E sections. H&E staining images with no significant inflammation or damage certificated the safety of the vaccine. The biochemical and hematological indicators with no difference between groups were all within the normal range (Fig. 7D and E). These above results showed that CDP-DFNS possesses good biosafety and meet the safety requirement of the oral vaccine.

## 4 Conclusion

In conclusion, this present study reported the feasibility of CDP-DFNS as oral vaccine delivery system. The CDP-DFNS carrier shows excellent physiochemical properties and transepithelial permeability. The results confirmed that CDP-DFNS facilitate the cellular uptake and transmembrane transport of antigens, further target to MLNs, and directly promoted the activation of DCs *in vitro*. More importantly, CDP-DFNS generate a more effective systemic and mucosal immune responses in vivo. Thus, we hope to provide a reference for the delivery system of oral vaccine with these specific advantages of CDP-DFNS.

## CRediT authorship contribution statement

**Jin He:** Writing – original draft, Supervision, Project administration, Methodology, Investigation, Data curation, Conceptualization. **Tianyu Zhu:** Methodology, Conceptualization. **Lin Yu:** Writing – review & editing, Methodology, Investigation. **Ningning Mao:** Investigation. **Xuanqi Lu:** Supervision, Investigation. **Xiaofeng Shi:** Supervision. **Xiangwen Deng:** Investigation. **Yang Yang:** Supervision. **Deyun Wang:** Funding acquisition.

## Declaration of competing interest

No potential conflicts of interest were reported by the authors in this work.

## Data Availability

The data that has been used is confidential.
