# Supplementary material for: A based Cistanche deserticola polysaccharide functional-nanoparticle delivery system for effective oral vaccine to facilitate both systemic and mucosal immunity through enhancing oral delivery
Source: Mater Today Bio. 2025 May 31;32:101939. doi: 10.1016/j.mtbio.2025.101939 (PMC12171821; doi:10.1016/j.mtbio.2025.101939)
Supplement: Multimedia component 1 [file mmc1.docx]

**Supporting Information**

***A nanoparticle system designed for effective delivery of oral vaccine enhancing both systemic and mucosal immunity***

Jin He^a^, Tianyu Zhu^a^, Lin Yu^a^, Ningning Mao^a^, Xuanqi Lu^a^, Xiaofeng Shi^a^, Xiangwen Deng^a^, Yang Yang^a^, Deyun Wang^a*^

a: College of Veterinary Medicine, Nanjing Agricultural University, Nanjing 210095, PR China.

* Correspondence and reprint requests: Deyun Wang, Ph.D.; Institute of Traditional Chinese Veterinary Medicine, College of Veterinary Medicine, Nanjing Agricultural University, Nanjing 210095, P R China; E-mail: dywang@njau.edu.cn; Tel: 0086-25-84395203; Fax: 0086-25-84398669.

**Figure S1.**
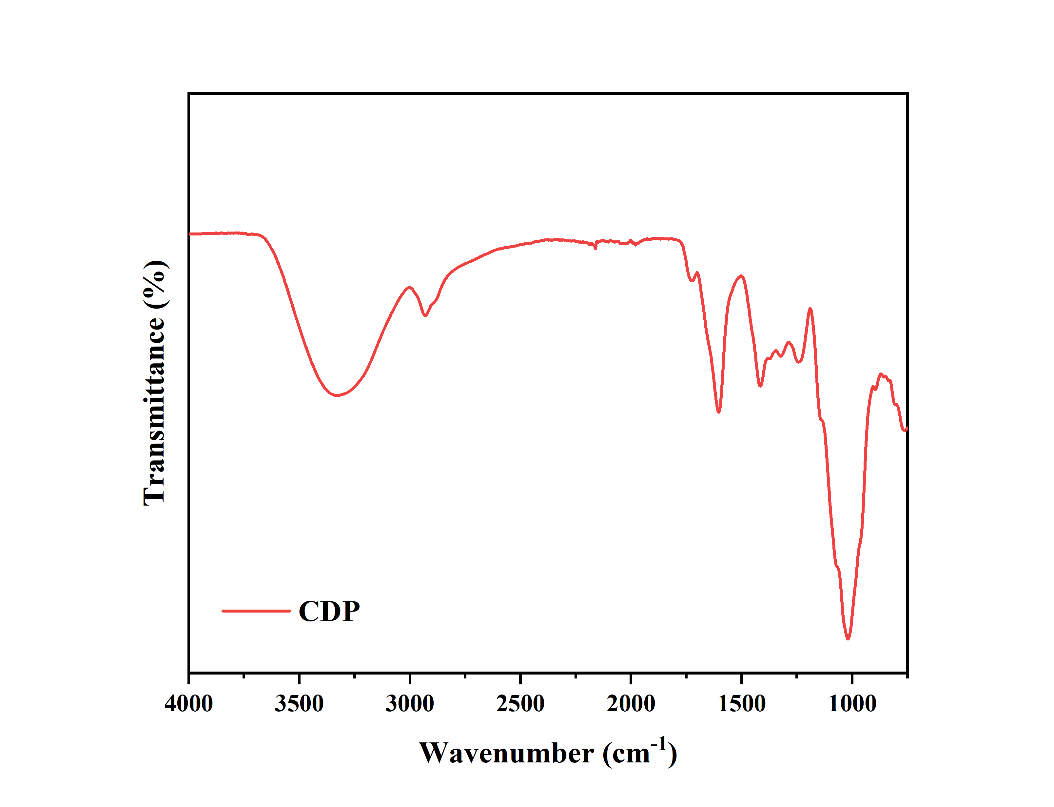
**The FTIR spectra of CDP.**


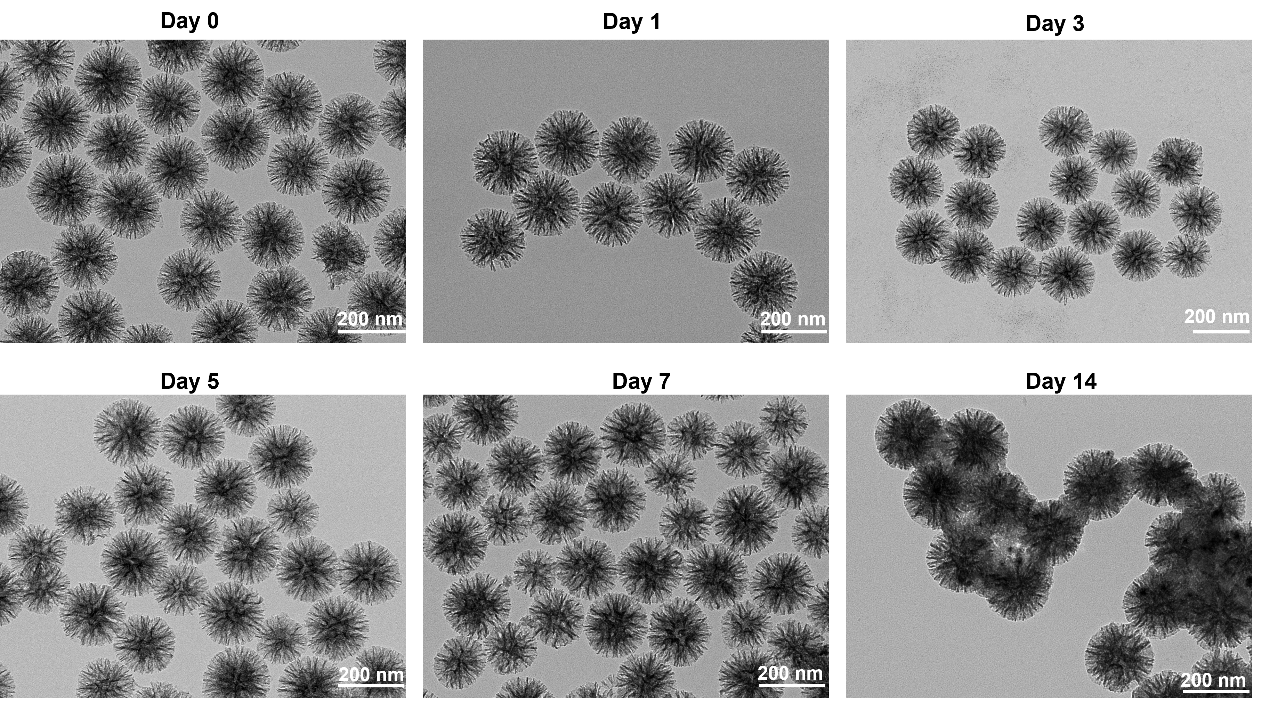


**Figure S2. TEM images of CDP-DFNS degraded in simulated body fluid. Scale bars, 200 nm.**

**
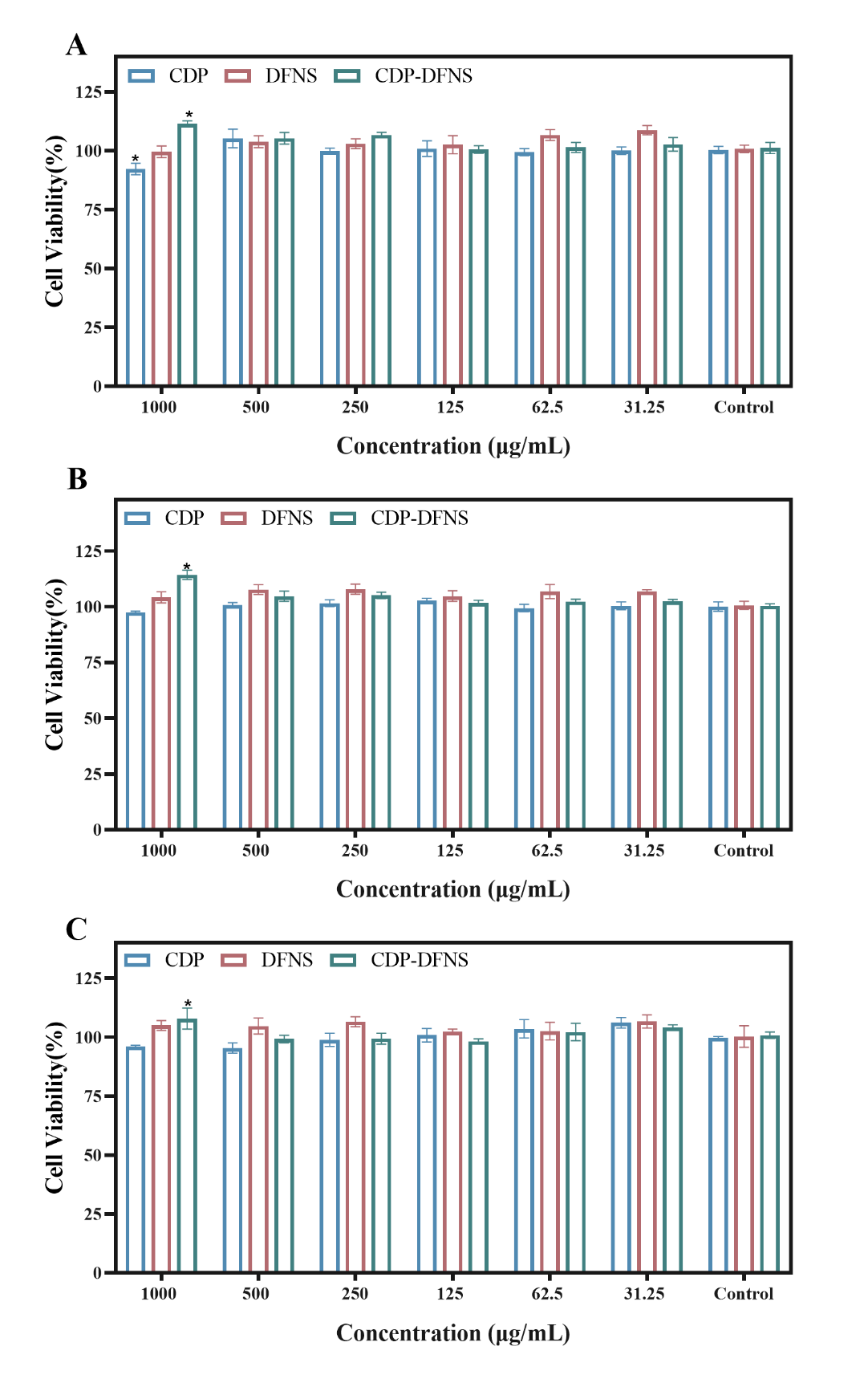
Figure S3. The cell viability of CDP, DFNS, and CDP-DFNS with 48 h (A), 24 h (B), and 4 h (C) co-incubation. Results were expressed as means ± SD, n = 4, *p < 0.05, **p < 0.01, ***p < 0.001.**

**
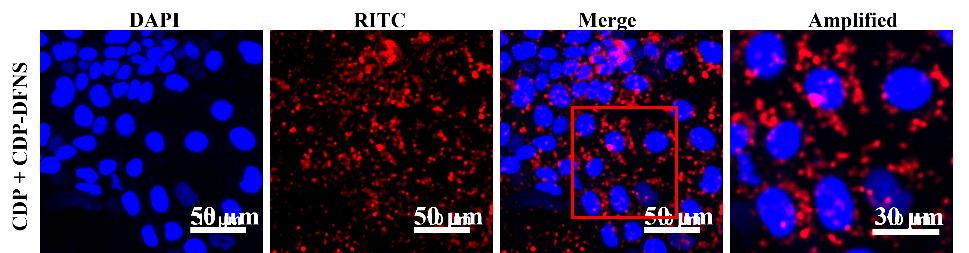
Figure S4. CLSM images of the cellular uptake of CDP + CDP-DFNS, red: RITC-NPs, blue: nuclei of the cells, scale bar 50 μm.**

**
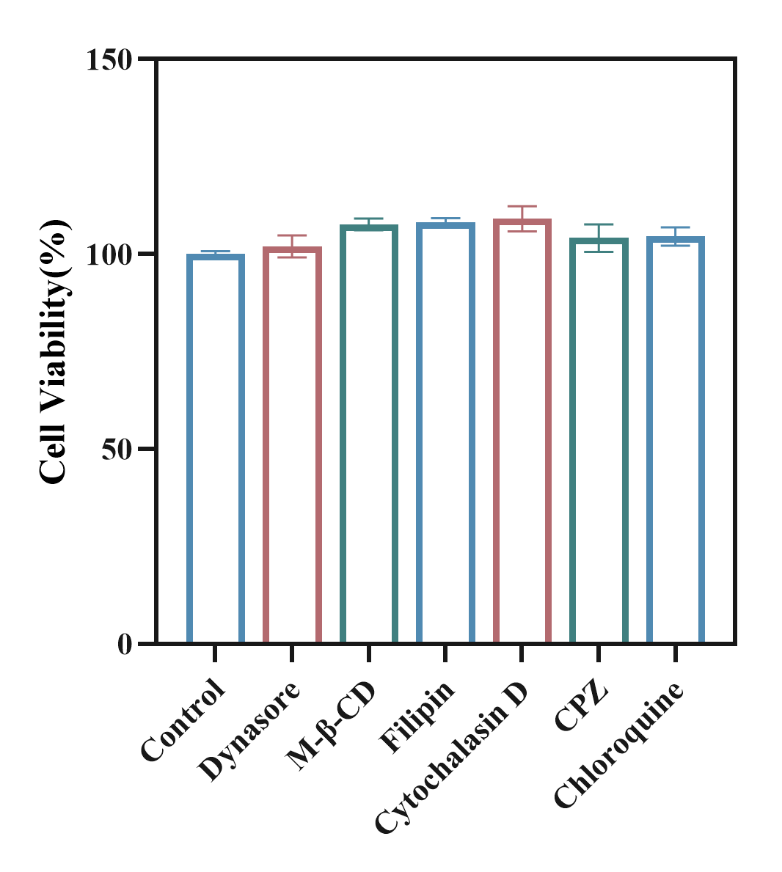
**

**Figure S5.** **The cell viability of endocytosis inhibitors with 30 min co-incubation.**


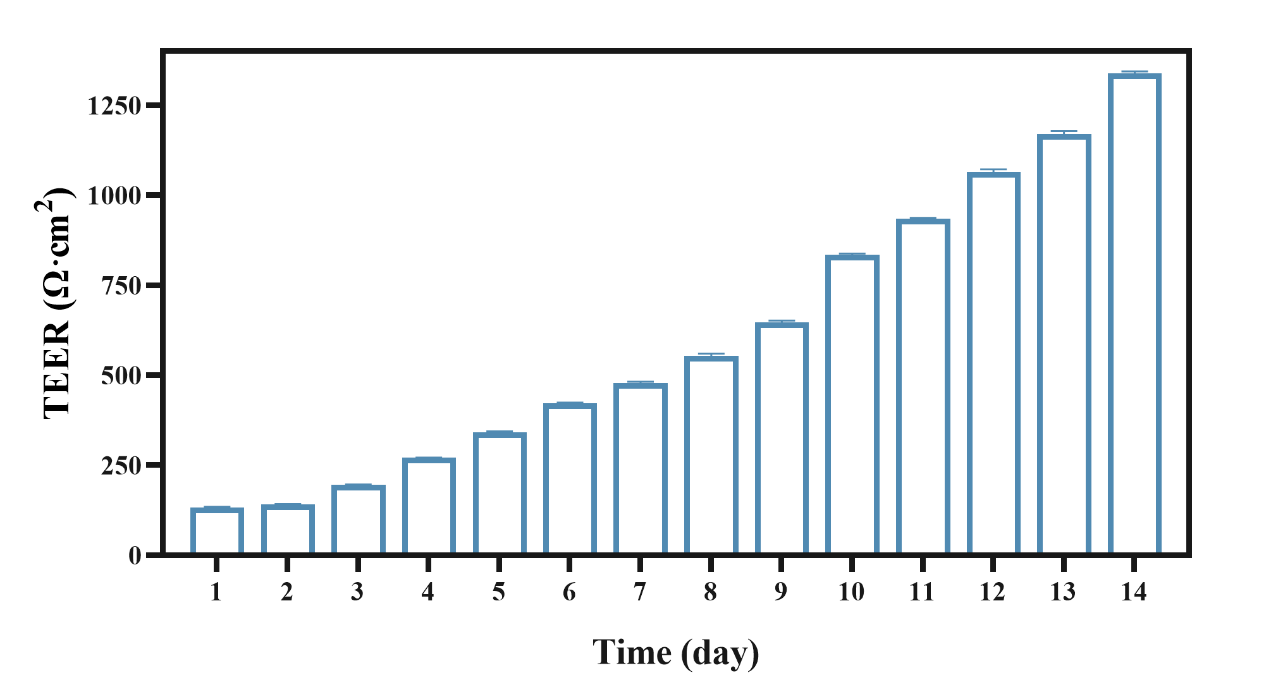
**Figure S6. The TEER value of caco-2 cells with continuous cultivation for 14 days. Results were expressed as means ± SD, n = 4.**

**
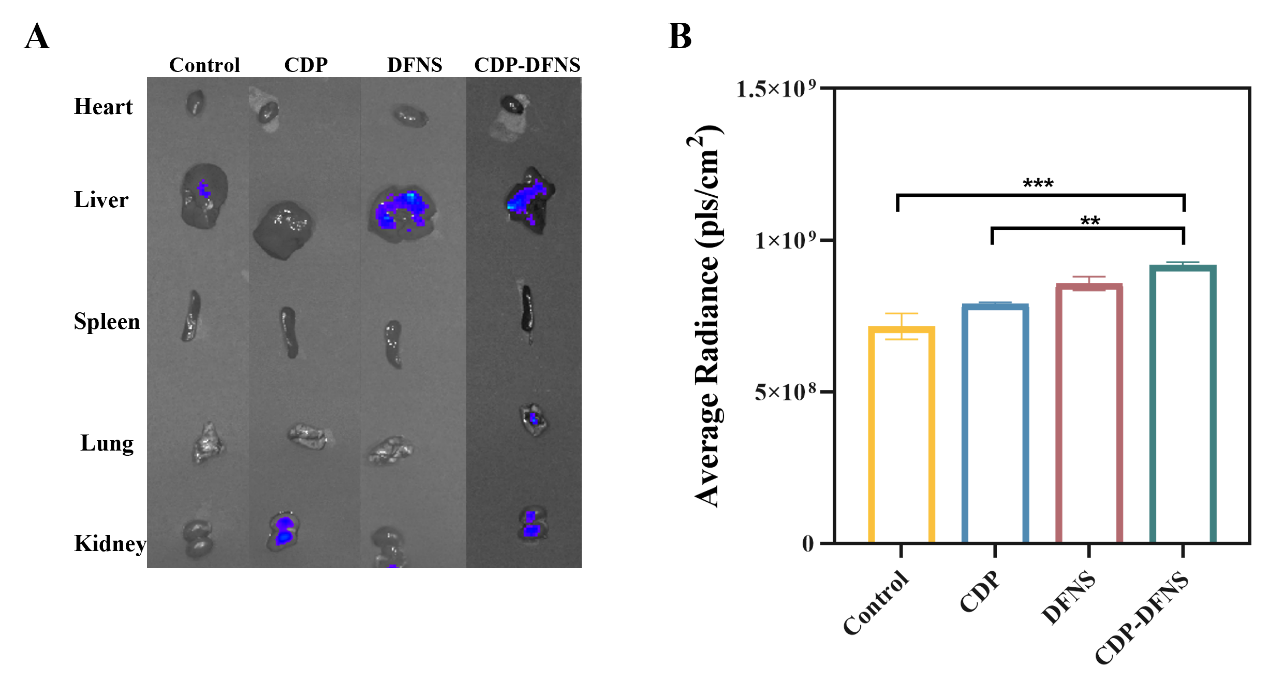
Figure S7. (A) Fluorescence images of Cy5.5-labeled antigen distribution in major organs (). (B) Quantitative analysis of Cy5.5-labeled antigen in major organs. Results were expressed as means ± SD, n = 3, *p < 0.05, **p < 0.01, ***p < 0.001.**

**
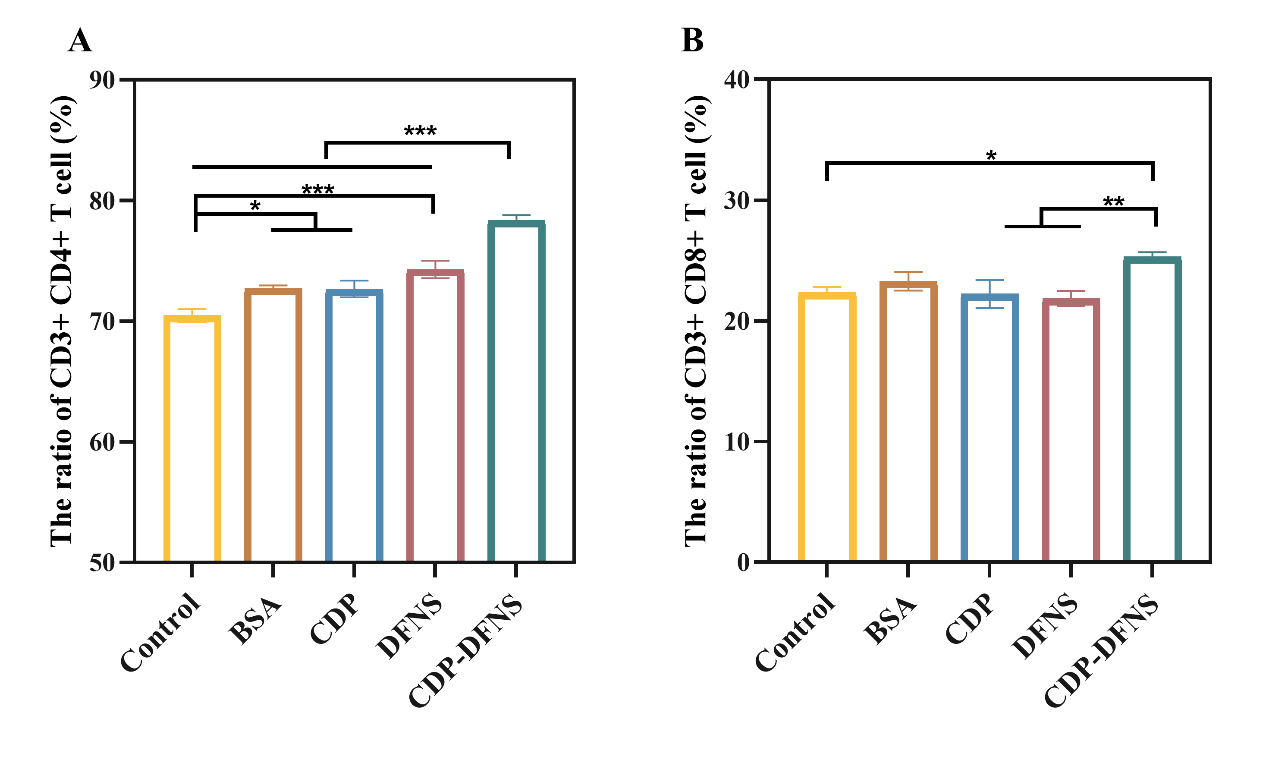
F****igure S8. The ratio ofCD3+ CD4+ T cells (A) and CD3+ CD8+ T cells (B) in spleen on day 28.**

**
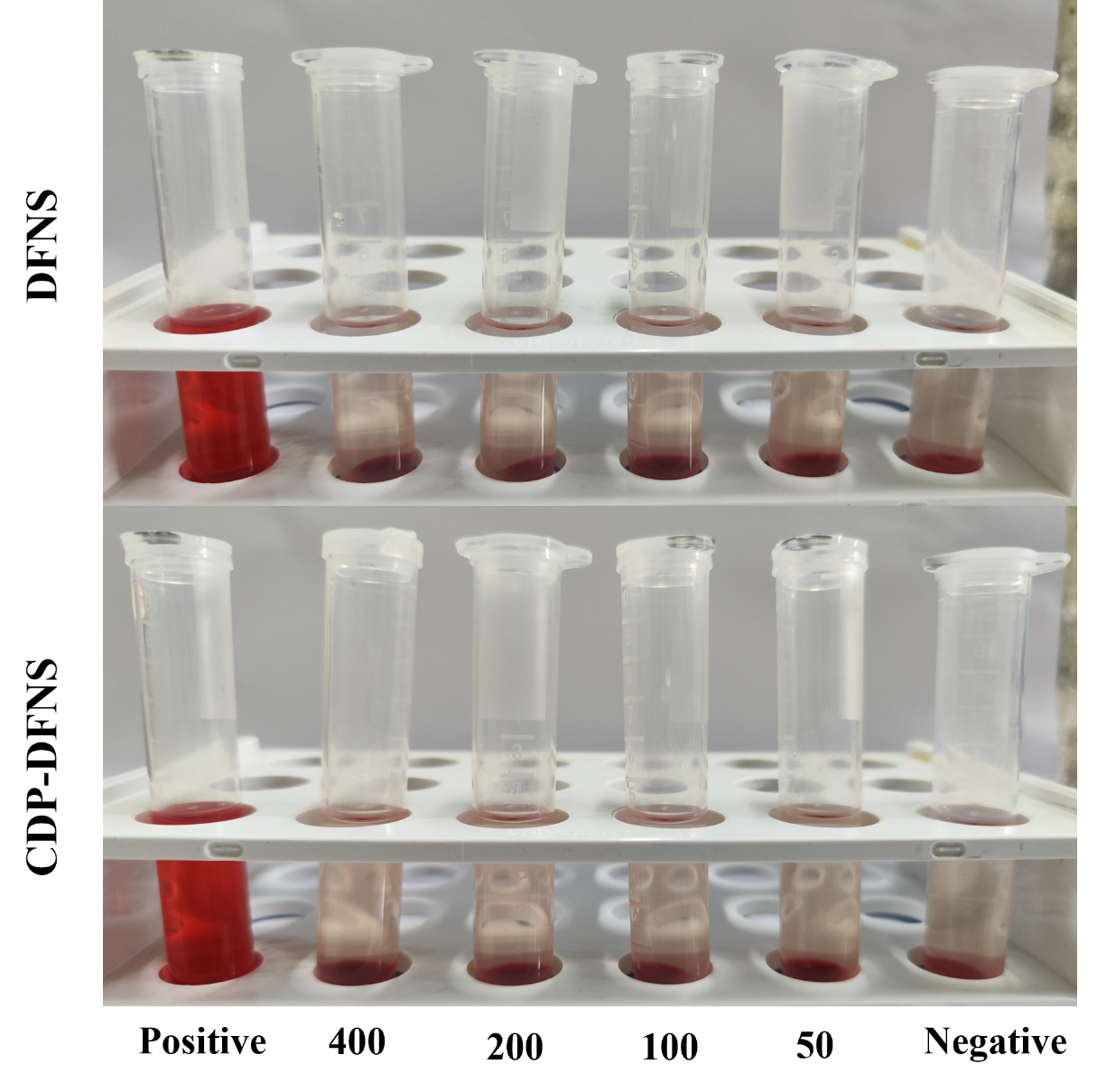
Figure S9. Hemolytic photographs DFNS and CDP-DFNS in concentration of 50-400 μg/mL.**

Table S1. The molecular parameters of CDP.

| Molecular  Characteristics | Parameter | Detection Results | Uncertainty |
| --- | --- | --- | --- |
| Molar mass moments | *Mn* | 50.334 kDa | 0.12773 |
|  | *Mp* | 62.846 kDa | 0.03900 |
|  | *Mw* | 132.124 kDa | 0.04060 |
|  | *Mz* | 448.870 kDa | 0.07891 |
|  | Polydispersity (Mw/Mn) | 2.625 | 0.13403 |

Table S2. The size, zeta potential, and PDI of DFNS-NH_2_.

| nanoparticles | Size (nm) | Zeta potential (mV) | PDI |
| --- | --- | --- | --- |
| DFNS-NH_2_ | 202.37±1.16 | 7.07±0.61 | 0.23±0.039 |
